# Influenza A Viruses Replicate Productively in Mouse Mastocytoma Cells (P815) and Trigger Pro-inflammatory Cytokine and Chemokine Production through TLR3 Signaling Pathway

**DOI:** 10.3389/fmicb.2016.02130

**Published:** 2017-01-12

**Authors:** Di Meng, Caiyun Huo, Ming Wang, Jin Xiao, Bo Liu, Tangting Wei, Hong Dong, Guozhong Zhang, Yanxin Hu, Lunquan Sun

**Affiliations:** ^1^Key Laboratory of Animal Epidemiology and Zoonosis of Ministry of Agriculture, College of Veterinary Medicine, China Agricultural UniversityBeijing, China; ^2^Key Laboratory of Veterinary Bioproduction and Chemical Medicine of the Ministry of Agriculture, Zhongmu Institutes of China Animal Husbandry Industry Co., LtdBeijing, China; ^3^Beijing Key Laboratory of Traditional Chinese Veterinary Medicine, Beijing University of AgricultureBeijing, China; ^4^Center for Molecular Medicine, Xiangya Hospital, Central South UniversityChangsha, China

**Keywords:** influenza A viruses, mast cells, pro-inflammatory cytokines, chemokines, TLR3 pathway

## Abstract

The influenza A viruses (IAVs) cause acute respiratory infection in both humans and animals. As a member of the initial lines of host defense system, the role of mast cells during IAV infection has been poorly understood. Here, we characterized for the first time that both avian-like (α-2, 3-linked) and human-like (α-2, 6- linked) sialic acid (SA) receptors were expressed by the mouse mastocytoma cell line (P815). The P815 cells did support the productive replication of H1N1 (A/WSN/33), H5N1 (A/chicken/ Henan/1/04) and H7N2 (A/chicken/Hebei/2/02) *in vitro* while the *in vivo* infection of H5N1 in mast cells was confirmed by the specific staining of nasal mucosa and lung tissue from mice. All the three viruses triggered the infected P815 cells to produce pro-inflammatory cytokines and chemokines including IL-6, IFN-γ, TNF-α, CCL-2, CCL-5, and IP-10, but not the antiviral type I interferon. It was further confirmed that TLR3 pathway was involved in P815 cell response to IAV-infection. Our findings highlight the remarkable tropism and infectivity of IAV to P815 cells, indicating that mast cells may be unneglectable player in the development of IAV infection.

## Introduction

Influenza A virus (IAV) is one of the most common respiratory pathogen in humans and animals. Since the first outbreak in Hong Kong in 1997, the highly pathogenic avian influenza (HPAI) H5N1 virus has become a public health threat due to its potential to cause serious illness and death in humans (Uyeki, 2009). Virus-induced acute lung injury or its more severe form, acute respiratory distress syndrome (ARDS), is a major cause of mortality by pandemic influenza and HAPI H5N1 infections; however, the exact mechanism of this injury is not fully understood. Several studies suggest that the main contributing factor is an increased production of inflammatory cytokines or “cytokine storm" (Thuy et al., 2011). This response may result from each individual cell producing more cytokines, or through chemokines-induced recruitment of a greater number of innate immune cells into the lung (Teijaro et al., 2011). Thus, the cellular sources involved in the resulting cytokine storm remain undetermined.

Mast cells are enriched near epithelial surfaces exposed to the external environment, and thus function as sentinels in the defense against host infection, they also play a role in initiating adaptive immune responses (Shelburne and Abraham, 2011). These processes are aided by the expression of a unique ‘armamentarium’ of receptor systems and mediators for responding to pathogen-associated signals (Marshall, 2004). Mast cells are crucial for optimal immune responses against bacterial, parasitic, and viral infections (Marshall, 2004). It was well demonstrated that mast cells played important roles in the pathogenesis of some viral infections, such as HIV-1, dengue virus, cytomegalovirus and bovine respiratory syncytial virus (Gibbons et al., 1990; King et al., 2000; Jolly et al., 2004; Sundstrom et al., 2004; Shirato and Taguchi, 2009). We previously demonstrated that mast cells were activated by H5N1 virus infection and escalated lung injury (Hu et al., 2012). However, it remains to be determined how this response is initiated, and whether IAV can infect and efficiently replicate in mast cells.

Influenza viruses bind to neuraminic acids (sialic acids, SA) on the surface of cells to initiate infection and replication (Knipe et al., 2001). The expression of SA linkages is cell type specific. For example, α-2,3-SA receptors are detected specifically in ciliated cells, while α-2,6-SA receptors are exclusively present in non-ciliated cells (Matrosovich et al., 2004). Historically, α-2,6-linked SA receptors, which are preferentially recognized by human influenza viruses, are detected exclusively in cells of upper respiratory tract of humans. However, both the α-2,6- linkages and α-2,3- linkages are present in the human lower respiratory tract, predominantly recognized by human and avian influenza viruses respectively (Raman et al., 2014). Thus, the type and distribution of SA is an important determinant of influenza virus tropism and pathogenesis (Suzuki et al., 2000); yet, little is known about SA receptor expression on mast cells.

During IAV infection, influenza viral dsRNA is sensed through several classes of pattern-recognition receptors (PRRs) including Toll-like receptors (TLRs) and retinoic acid-inducible gene-I-like receptors (RLRs) (Yu and Levine, 2011). Among the PPRs, toll-like receptor 3 (TLR3) and cytolytic RNA helicases retinoic acid-inducible gene I (RIG-I) are the most common transducers of viral dsRNA signals. Once stimulated with their respective agonist, TLR3 recruits adaptor molecule TIR-domain containing adaptor inducing interferon-β (TRIF), while RIG-I associated with mitochondrial antiviral signaling (MAVS) to initiate downstream signaling. Both these pathways activate the transcription factor nuclear factor (NF)-kB, leading to the production of inflammatory cytokines, chemokines, and activation of interferon regulatory factor (IRF) 3 and/or 7 to induce the key antiviral mediator type I IFNs (Takeuchi and Akira, 2009). Recently, Le Goffic et al. (2007) demonstrated the importance of TLR3 in the inflammatory cytokine response to IAV in lung epithelial cells *in vitro* (Le Goffic et al., 2007). IAV-TLR3 interactions are also critical for viral pathology *in viv*o. Previous studies showed that the association between RIG-I and viral ssRNA bearing an uncapped 5′-triphosphate end (Pichlmair et al., 2006) and this association resulted in the production of IFNs (Kato et al., 2006). Moreover, RIG-I played a key role in the expression of proinflammatory cytokines in mast cells infected by IAV. However, the role of TLR3 during IAV infection in mast cells remains unexplored.

Therefore, in the present study we sought to determine the presence and role of SA receptors on mouse mastocytoma cell line (P815). We demonstrated that P815 cells expressed both α-2, 3-, and α-2, 6- linked SA receptors to initiate IAV infection. In addition, P815 cells supported productive replication of IAVs *in vitro* while the *in vivo* infection of H5N1 in mast cells was confirmed by the specific staining of nasal mucosa and lung tissue from mice. Following IAV infection, P815 cells mediated substantial hyper-induction of pro-inflammatory cytokines and chemokines, and TLR3 signal pathways probably involved in the process. This provides insight for the development of novel strategies to combat influenza infection by targeting mast cells.

## Materials and Methods

### Ethics Statement

All mouse experimental protocols complied with the guidelines of the Beijing Laboratory Animal Welfare and Ethics Committee, and were approved by the Beijing Association for Science and Technology (the approval ID is SYXK-2009-0423). All experiments with live H5/H7 subtype viruses were performed in a biosafety level 3 containment laboratory (the approval number is CNAS BL0017) approved by the Ministry of Agriculture of the People’s Republic of China.

### Viruses and Cells Culture

The avian influenza viruses H5N1 (A/Chicken/Henan/1/04) (Hu et al., 2012) and H7N2 (A/Chicken/Hebei/2/02) were isolated from infected chicken flocks, and propagated in the allantoic cavities of 10-day-old embryonated chicken eggs for 24–48 h at 37°C. The working stocks of human influenza virus H1N1 (A/WSN/33) were generated in MDCK cells. Virus titers were determined by standard plaque assays. The 50% lethal dose (LD_50_) in mice was determined as previously described (Hu et al., 2012). The mouse mastocytoma cell line P815 and the Madin-Darby canine kidney cell line MDCK were cultured as previously described (Hu et al., 2012).

### *In vitro* Viral Infection and LE-PolyI:C Treatment

Cell monolayers were formed in tissue culture plates by seeding 6-well (1 × 10^6^ cells/well) or 12-well (5 × 10^5^ cells/well) plates, washed with DMEM and infected with viruses at a multiplicity of infection (MOI) of 0.1 for 1 h at 37°C. After incubation, cells monolayers were washed and DMEM supplemented with 1% bovine serum albumin was added to each well and incubated for the indicated times. Polyinosine-polycytidylic acid (polyI:C), a synthetic mimic of viral double-stranded RNA, was used as a positive control. Liposome-encapsulated PolyI:C (LE-PolyI:C) used in this study was prepared as described previously (Wong et al., 1999), diluted to a final concentration of 10 μg/ml and incubated with cells at 37°C for the indicated times.

### *In vivo* Viral Challenge

Female BALB/c mice (8–10 weeks) were purchased from Vital River Laboratories (Beijing, China), and feed pathogen-free food and water in independent ventilated cages. Mice were first anesthetized with Zotile^®^ (Virbac, Carros, France), and then infected intra-nasally with PBS-diluted H5N1 virus (5LD50) or PBS alone. The nose and lung tissues were then collected 6 days post-infection.

### Immunofiuorescence Staining and Confocal Microscopy

Tissue samples were fixed in 4% neutral formalin, embedded in paraffin, and serially cut at a thickness of 5 μm. Cultured cells were fixed on a polylysine-coated slide with 4% formaldehyde, and blocked with 3% BSA. To visualize surface receptors, slides containing fixed tissues or cells were directly stained with fluorescein- *Sambucus nigra* bark lectin (SNA, specific to SAα2,6-Gal) or fluorescein- *Maackia amurensis* lectin I (MAA-I, specific to SAα2,3-Galβ(1-4)GlcNAc). To confirm the specificity of lectin binding, monolayers were washed and treated with 250 mU/ml of neuraminidase from *Clostridium perfringens* (New England BioLabs, Beijing, China) for 3 h prior to lectin staining. To detect tryptase expression or IAV nucleoprotein (NP) antigen, cells were permeabilized with 0.5% Triton X-100 before blocking, then tissue sections or cell slides were either incubated with a rabbit anti-mast cell tryptase monoclonal antibody (Abcam, [EPR8476], ab134932) for 2 h at room temperature, or a mouse anti-IAV NP monoclonal antibody (Abcam [AA5H], ab20343) at 4°C overnight. After washing three times with PBS-T, tissue sections were further incubated with a Texas red-conjugated goat anti-mouse or rabbit secondary antibody, and cell slides were incubated with a FITC-conjugated goat anti-mouse secondary antibody (Abcam) for 1 h at room temperature. To visualize the nuclei, all slides were stained with 3 μg/ml 4′,6′-Diamidine-2-phenylindole (DAPI) (Sigma–Aldrich) for 5 min at room temperature and then examined under a laser scanning confocal microscope (Leica TCS SP5 II, Leica Microsystems, Wetzlar, Germany).

### Flow Cytometry Analysis

Cultured P815 cells (1 × 10^6^) were pelleted, washed twice with DMEM, once with flow buffer (PBS with 2% FBS) and then re-suspended in 200 μl of fluorescein- SAA or fluorescein- MAA I at different dilutions. The cells were incubated for 1 h at 4°C, then washed and re-suspended in flow cytometry buffer for analysis on a BD FACSCalibur using Cell Questpro software (BD Biosciences, California).

### Transmission Electronmicroscopy (TEM)

Cells were trypsinized and fixed using 2.5% (v/v) glutaraldehyde in PBS for 2 h at 4°C. Cells were then washed with PBS, post-fixed in 1% osmium tetroxide, and washed and dehydrated in series of ethanol solutions. The dehydrated pellets were embedded in epoxy resin, and 70-nm sections were cut. Then the sections were placed on copper sieves and stained with uranyl acetate and lead citrate. Images were obtained using a JEM-1230 TEM (JEOL, Japan Electronics Co., Ltd, Tokyo, Japan).

### Real-Time Quantitative PCR

Total RNA was extracted from cells in Trizol reagents (Invitrogen, Carlsbad, CA, USA). DNase I-treated RNA (0.2 μg) was reverse transcribed into cDNA using random or universal primers for IAV (Uni 12) (Hoffmann et al., 2001) with an EasyScript First-Strand cDNA Synthesis Super Mix (TransGen Biotech, China) according to the manufacturer’s instruction. Reactions were performed in triplicate using a Power SYBR^®^ Green PCR Master Mix (Applied Biosystems, Warrington, UK) and the Applied Biosystems 7500 system. The mRNA expression levels of the genes were normalized to β-actin, compared with mock-infected cells, and quantified by the 2^-ΔΔCT^ method. The sequences of the primers are listed in Supplementary Table S1. The amplifications were performed as follows: a 10 min hot start at 95°C, followed by 40 cycles of denaturation at 95°C for 15 s, annealing at 55°C for 35 s, and extension at 72°C for 40 s.

### Proteome Profiler Antibody Array Assay

Cell-free supernatants were acquired by centrifugation, then the levels of cytokines and chemokines were analyzed by the Mouse Cytokine Array Panel A (R&D Systems, Minneapolis, MN, USA) according to the manufacturer’s instructions, which could provide parallel determination of the relative levels of 40 kinds of selected mouse cytokines and chemokines.

### Cytokine and Chemokine Quantification

The concentration of IL-6, IFN-γ, RANTES, IP-10, IFN-α, IFN-β, and TNF-α in the supernatant of cell cultures was determined using ELISA kits (eBioscience, San Diego, CA, USA) according to the manufacturer’s instructions.

### Co-immunoprecipitation

Cells were either mock-treated, LE-PolyI:C-treated, or infected with IAV viruses at a MOI of 0.1. At 6 h post-infection, the cells were washed with cold PBS and lysed for 15 min on ice with RIPA lysis buffer containing 50 mM Tris-HCl (pH7.4), 150 mM NaCl, 1% NP40, 0.25% sodium deoxycholate and a protease inhibitor cocktail (Beyotime Institute of Biotechnology, Beijing, China). Lysed cells were pelleted and the supernatants were incubated with the indicated antibodies (anti-TLR3 antibody or an isotype IgG antibody from Abcam) for 2 h at 4°C with gentle shaking. The samples were then added to a preprocessed EZview^TM^ Red Protein A/G Affinity Gel (Sigma) and incubated for 6 h. After washing three times with lysis buffer, the beads were boiled in SDS loading buffer, and then analyzed by immunoblot with the indicated antibodies (anti-TLR3 antibody or anti-TRIF antibody from Abcam).

### Western Blot

Cell lysates were prepared using lysis buffer as described above, and protein concentrations were determined using a BCA protein assay kit (Beyotime Institute of Biotechnology). Equal amounts of protein were separated by SDS-PAGE and transferred to a polyvinylidene difluoride (PVDF) membrane (Millipore, Beijing, China). The membranes were blocked using 5% non-fat dry milk (BD Biosciences) at room temperature for 2 h, and then incubated overnight at 4°C with antibodies (anti-TLR3 antibody and anti-TRIF antibody from Abcam; Influenza A NS1 antibody from Santa Cruz Biotechnology, Dallas, Texas, USA). After three 10 min washes in Tris-buffered saline containing 0.05% Tween (TBST), the membranes were incubated for 1 h at room temperature with the appropriate horseradish peroxidase-conjugated secondary antibody. Protein bands were visualized using the Western Lightning Plus-ECL (Perkin Elmer, MA, USA). β- Actin was used as a loading control.

### Inhibition of TLR3 Activation Using Specific Inhibitions

P815 cell monolayers were pre-incubated with TLR3/dsRNA complex inhibitor (Calbiochem, Darmstadt, Germany) at a concentration of 25 and 50 μM (diluted with DMSO) for 12 h. They were then were infected with virus at a MOI of 0.1, exposed to LE-PolyI:C, or mock treated as described above. The same concentration of inhibitors was immediately added after 1 h of viral incubation. Samples were collected 24 and 36 h after infection.

### Statistical Analysis

Statistical analysis was performed by one-way ANOVA using the SPSS software suite (version 12.0), and a *P*-value of <0.05 was considered statistically significant. Results were expressed as mean ± standard deviation (SD) of at least three independent experiments.

## Results

### P815 Cells Express Both α-2,3- Linked and α-2,6- Linked SA Receptors

**FIGURE 1:**
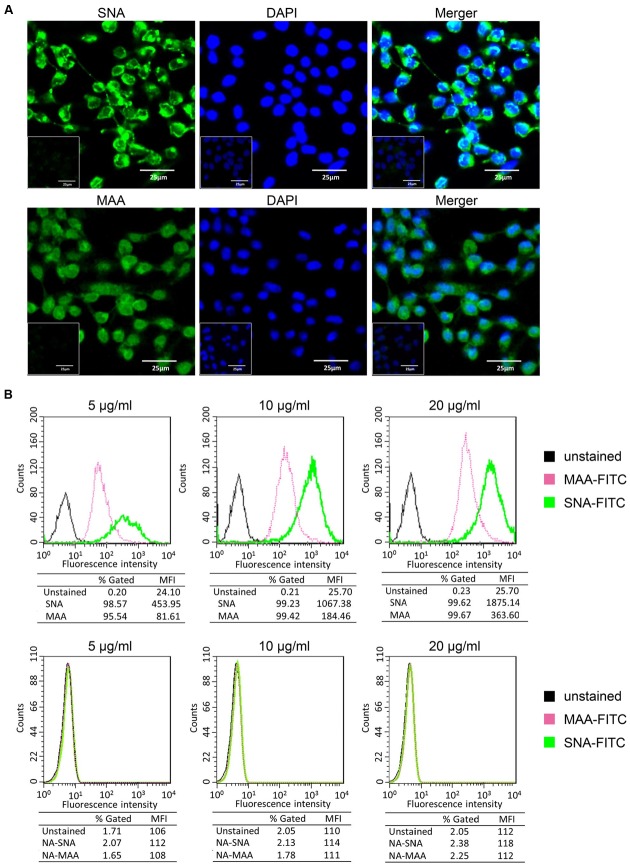
**P815 cells express α-2, 3- and α-2,6- linked sialic acid (SA) receptors. (A)** The mouse mastocytoma cell line P815 was placed on polylysine-coated slides and stained with FITC-conjugated SNA or MAA-I (green), and DAPI (blue) for nuclei. Image inserts depict cells pre-treated with neuraminidase to abolished sialic acid residue staining. **(B)** Trypsinized P815 cells were incubated with FITC-conjugated SNA or MAA-I (concentrations from left to right are 5, 10, and 20 μg/ml) and analyzed using flow cytometry to determine relative percentages of cells expressing α-2,3-SA (MAA, pink) or α-2,6-SA (SNA, green), compared to unstained cells (black). “NA-MAA” and “NA-SNA” indicated that P815 cells pre-treated with neuraminidase to abolished sialic acid residue staining. Results shown are representative of three independent experiments.

Influenza viral HA proteins initiate infection by interacting with sialic acid residues coating the surface of host cells. In general, human influenza viruses and *S. nigra* bark lectin (SNA) preferentially bind α-2,6- linked SA receptors, while avian influenza viruses and *M. amurensis* lectin (MAA) predominantly bind to α-2,3- linked SA receptors (Springer et al., 1969; Suzuki et al., 2000; Ibricevic et al., 2006; Shinya et al., 2006). Here we used the mouse mastocytoma cell line, P815, as a mast cell model. To determine the susceptibility of P815 cells to influenza viruses, we first analyzed the distribution of surface SA receptors by lectin staining as described in the materials and methods. Both α-2,3- and α-2,6- linked SA receptors were expressed on the surface of P815 mouse mastocytoma cells, with the intensities of SNA being visually stronger than one of the two isoforms of MAA (**Figure 1A**). We treated P815 cells with a broad-spectrum neuraminidase to cleave sialic moieties abolished lectin binding, and confirmed the specificity of SNA and MAA staining (**Figure 1A**, insets).

To quantitatively analyze the sialic acid residues, P815 cells were stained with different concentrations of FITC-conjugated lectins and assayed by flow cytometry. As shown in **Figure 1B**, at an lectin concentration of 5 μg/ml, 95.54% of P815 cells were detected positive expression of α-2,3-linked SA receptors (FITC-MAA) and 98.57% of α-2,6-linked receptors (FITC-SNA). At higher concentrations (10 and 20 μg/ml), almost all cells positively expressed both SA receptors (>99%). The mean fluorescence intensity (MFI) depended on the lectin concentration, and the MFI of SNA was significantly higher (>fivefold) than that of MAA at all the concentrations. These data suggested that the expression of α-2,6- linked SA receptors was more abundant on P815 mastocytoma cells than α-2,3- linked SA receptors.

Taken together, these data suggested that both α-2,3- and α-2,6- linked SA receptors were expressed on the surface of P815 mouse mastocytoma cells.

### P815 Cells Support the Replications of IAVs

We previously demonstrated that H5N1 infection could activate mast cells (Hu et al., 2012). To determine if IAV could enter and replicate productively in mastocytoma cells, we examined the replication kinetics of human and avian IAVs in P815 cells. As shown in Supplementary Figure S1, all three subtypes of IAVs productively replicated in P815 cells as measured by hemagglutination assay (left), plaque formation (middle), and viral RNA expression (right). In P815 cells, the replications H1N1 and H5N1 viruses were more efficient than H7N2 virus. These data indicated that IAVs replicated well in P815 cells with some degree of tropism selectivity.

**FIGURE 2:**
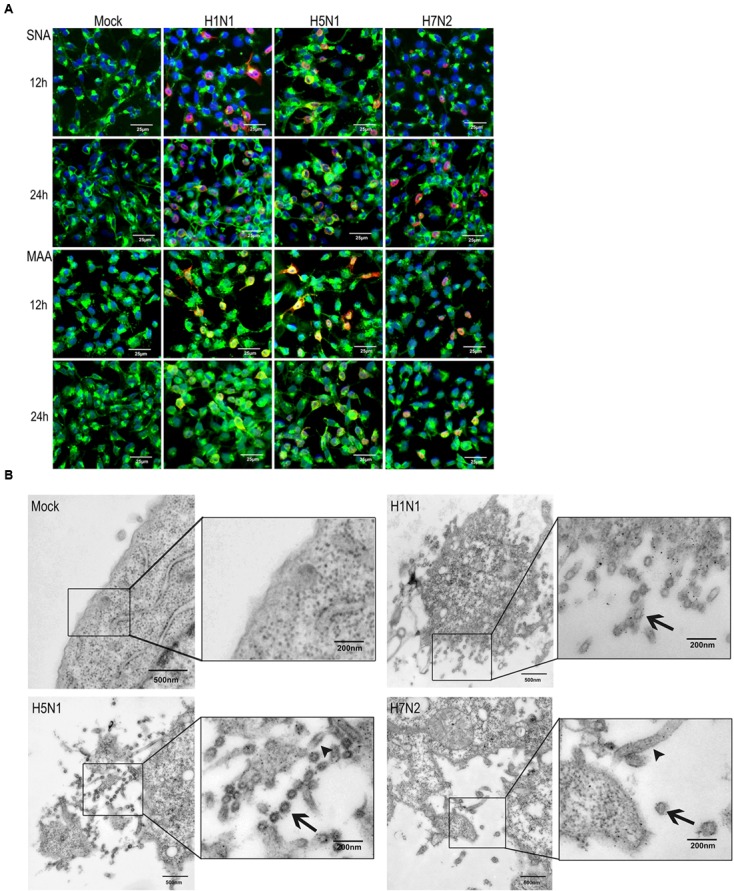
**Influenza viruses can infect P815 cells *in vitro***. P815 cells were mock-treated or infected with three subtypes of influenza viruses at a MOI of 0.1. **(A)** Immunofluorescence detection of viral nucleoprotein (NP) antigen and SA receptors in P815 cells. At the indicated times post-infection, cells were fixed and stained for α-2,6- or α-2,3- sialic acids (green) and influenza NP (red). Results shown are representative of three separate experiments. **(B)** Transmission electron microscopy of influenza viruses released from the cell surface. Higher magnifications are in the boxes on the right. Arrows denote the virus particles.

To corroborate this finding, P815 cells were co-stained with α-2,3- or α-2,6- linked SA receptors and viral NP (**Figure 2A**). The wide distribution of α-2,6- linked SA receptors was consistent the effective replication of H1N1. In addition, a large number of NP-positive cells were observed when infected with H5N1, but were less abundant in H7N2 infected cells, which was consistent with the viral replication profiles (Supplementary Figure S1). To further validate the permissiveness of P815 for IAVs replication, we used transmission electron microscopy. As shown in **Figure 2B**, budding virions were present on the surface of cells infected with each of the three virus subtypes. Moreover, many viral particles were found to be associated with the surface of the cells infected with H1N1 and H5N1, but were less obvious on H7N2 infected cells. Together, the above data suggested that IAVs could bind and enter into P815 mastocytoma cells, where the efficient replication was supported.

**FIGURE 3:**
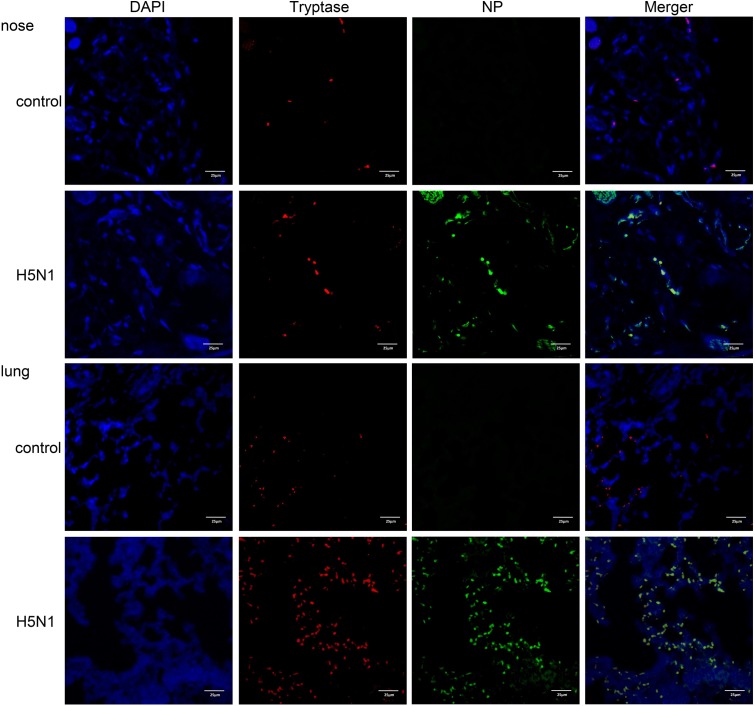
**H5N1 influenza virus can infect mouse mast cells *in vivo***. Representative nasal mucosa and lung sections from control or H5N1 virus-infected mice. Sections were analyzed by immunofluorescence staining (Blue = nuclei, red = tryptase, and green = influenza NP). Results shown are representative of three independent repeats.

To determine if IAVs can infect mast cells *in vivo*, we used a mouse H5N1 virus infection model. Mice were either infected with H5N1 or PBS and the nasal mucosa and lung tissues were resected and analyzed by immunofluorescence. Cells double positive for the virus-specific antigen NP and the mast cell specific protein tryptase were present in H5N1 infected mice but not in PBS treated mice (**Figure 3**). These data suggested that IAVs probably could actively infect mast cells *in vivo*.

### IAVs Induce Cytokine and Chemokine Production in P815 Cells

**FIGURE 4:**
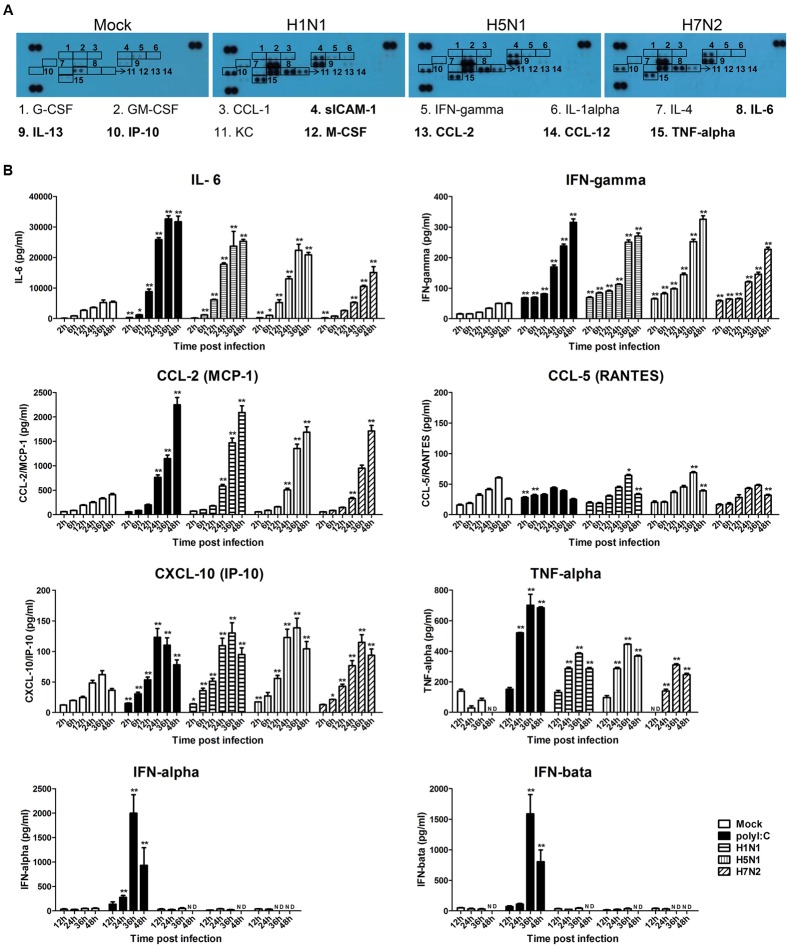
**Influenza virus infection causes the release of pro-inflammatory cytokines and chemokines. (A)** P815 cells were infected with H1N1, H5N1, and H7N2 at a MOI of 0.1, or mock treated for 24 h. Cell-free supernatants were then analyzed for cytokine and chemokine content using a cytokine array panel. Numbered boxes denote up-regulated expression, with the most dramatic increases annotated in bold. Results shown are representative of two separate experiments. **(B)** P815 cells were infected with H1N1, H5N1, and H7N2 at a MOI of 0.1, exposed to LE-PolyI:C, or mock treated. At the designated time points, cell supernatants were harvested and the expression of IL-6, IFN-γ, CCL-2, CCL-5, IP-10 TNF-α, IFN-α, and IFN-β was analyzed by ELISA. Graphs shown are mean ± SD of three independent replicates. Asterisks indicate statistically significant increases compared to mock treatment (^∗^*P* < 0.05, ^∗∗^*P* < 0.01). ND, not detectable.

Our previous data suggested that H5N1-activated mast cells could intensify lung injury by releasing the pro-inflammatory mediators including histamine, tryptase, and IFN-γ (Hu et al., 2012). To more specifically examine the cytokines and chemokines released by P815 cells upon avian and human IAVs infection, we performed an antibody array analysis. As shown in **Figure 4A**, the production of sICAM-1, IL-6, IL-13, IP-10, M-CSF, CCL-2, CCL-12, and TNF-α was augmented in the supernatants of P815 cells infected with all three viruses subtypes. The release of G-CSF, GM-CSF, CCL-1, IFN-γ, IL-1α, IL-4, and CXCL-1 was only moderately increased. To further confirm these findings, we used ELISA to conduct kinetic profiles of the selected cytokines and chemokines that were potentially involved in responses to influenza infection. The three virus subtypes, and LE-PolyI:C, induced significantly higher levels of IL-6 and IFN-γ compared to mock treated cells (**Figure 4B**). The production of CCL-2 did not occur until 12 h post-infection, but then increased from 24 to 48 h post-infection (**Figure 4B**), while the secretion of IP-10 and TNF-α increased gradually peaking at 36 h post-infection. In contrast, all three IAV subtypes induced a relatively low expression of CCL-5 for 24 h post-infection, but this increased at later time points. The expressions of antiviral cytokines IFN-α and IFN-β did not change from 2 to 48 h post-infection (**Figure 4B**).

To further analyze the expression kinetics of various cytokines and chemokines released by P815 cells infected with each of the three virus subtypes and LE-PolyI:C, we performed quantitative RT-PCR. The mRNA expression profiles of IL-6, IFN-γ, TNF-α, CCL-2, CCL-5, and IP-10 were similar to the data generated from ELISA (Supplementary Table S2). However, while the expression levels of a large number of pro-inflammatory cytokines and chemokines were up-regulated, the mRNA levels of the antiviral genes IFN-α, IFN-β and the anti-inflammatory cytokine IL-10 were unchanged during viral infection (Supplementary Table S2). Taken together, these data suggested that following IAV infection P815 cells released a range of pro-inflammatory cytokines and chemokines.

### TLR3 Plays a Key Role in the Expression of Proinflammatory Cytokines in P815 Cells Infected by IAV

**FIGURE 5:**
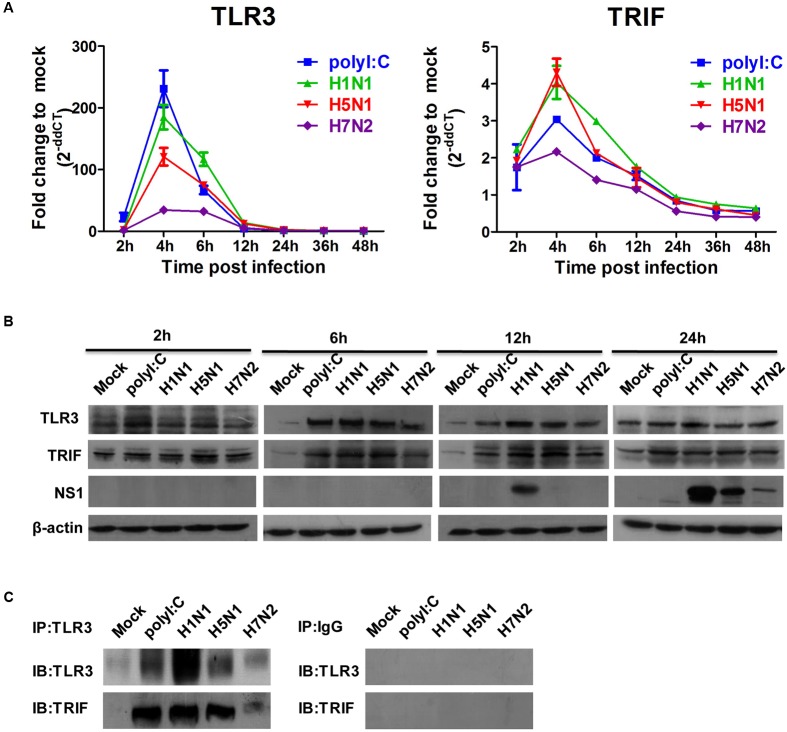
**Influenza A viruses up-regulates the expressions of TLR3 and TRIF mRNA and proteins**. P815 cells were treated or infected as described in **Figure 4B**. **(A)** Total RNA was isolated at the designed times and examined with quantitative real-time PCR. The expression of TLR3and TRIF is shown. The data are presented as the relative fold change over mock treatment, and are pooled from three independent experiments. **(B)** P815 cells were collected at 2, 6, 12, and 24 h post-infection or treatment, and then analyzed by immunoblotting for expression of TLR3, TRIF, viral NS1 protein and β-actin. Data are representative of three separate experiments. **(C)** Endogenous interactions of TLR3 with TRIF. Whole cell extracts at 6 h (TLR3-TRIF) post-infection were immunoprecipitated with the indicated antibodies or isotype IgG controls and analyzed by western blot analysis.

Given that IAVs could infect P815 cells and promote the release of inflammatory cytokines and chemokines, we next examined expression level of viral RNA sensor TLR3 involved in the transduction of inflammatory molecule signals. The mRNA expression level of TLR3 in P815 cells infected with IAVs peaked 4 h post-infections, and returned to baseline levels by 12 h post-infection (**Figure 5A**). In addition, the mRNA expression profiles of the adaptor molecules TRIF increased in a manner consistent with TLR3 levels; however, the fold increase compared to mock infected cells was much lower. Consistent with mRNA levels, the protein expression of TRL3 and TRIF was strongly augmented in IAV infected P815 cells (**Figure 5B**). To confirm if TLR3 were indeed activated, co-immunoprecipitation experiments were used to test the endogenous interactions of TLR3 with TRIF. Co-precipitation of TLR3/TRIF was evident in P815 cells infected with all three IAVs (**Figure 5C**). These data indicated that the viral RNA sensors TLR3 were expressed following IAV infection.

**FIGURE 6:**
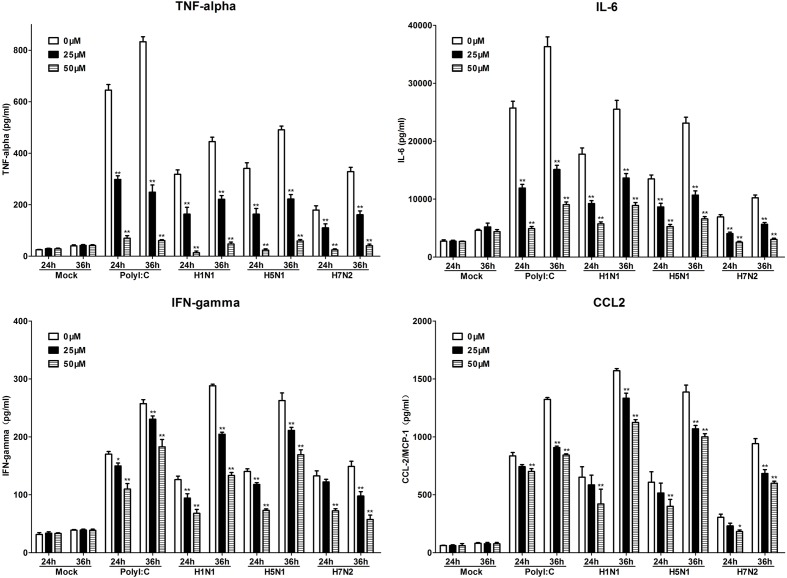
**Toll-like receptors/dsRNA complex inhibitor can reduce the releasing of proinflammatory cytokines in P815 cells infected by IAV**. P815 cells treatment with TLR3/dsRNA complex inhibitor were infected with H1N1, H5N1, and H7N2 at a MOI of 0.1, exposed to LE-PolyI:C, or mock treated. At the designated time points, cell supernatants were harvested and the expression of TNF-α, IL-6, IFN-γ, and CCL-2 was analyzed by ELISA. Graphs shown are mean ± SD of three independent replicates. Asterisks indicate statistically significant increases compared to mock treatment (^∗^*P* < 0.05, ^∗∗^*P* < 0.01).

**FIGURE 7:**
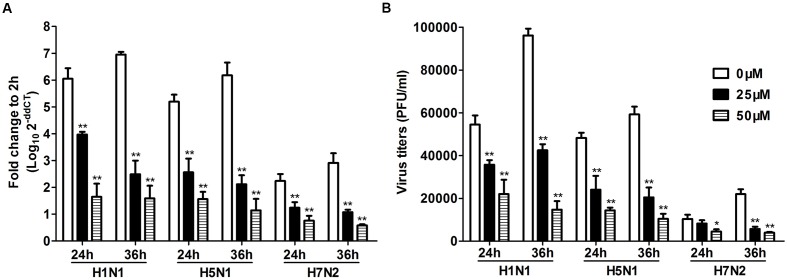
**Toll-like receptors/dsRNA complex inhibitor can decrease the viral loads of IAV in P815 cells**. P815 cells treatment with TLR3/dsRNA complex inhibitor were infected with H1N1, H5N1, and H7N2 at a MOI of 0.1, exposed to LE-PolyI:C, or mock treated. Cells were homogenized in Trizol and relative viral NS gene quantification was determined by real time PCR **(A)**. Culture supernatants were collected at the indicated times post-infection, and virus titers in the supernatants were determined by plaque assay **(B)**. Results shown are pooled from three independent repeats. Asterisks indicate statistically significant increases compared to mock treatment (^∗^*P* < 0.05, ^∗∗^*P* < 0.01).

To further investigate the role of TLR3 in P815 cells during IAV infection, we utilized a novel TLR3/dsRNA complex inhibitor to disrupt the interaction between IAV and TLR3 in P815 cells. The effects of the inhibitors on the release of pro-inflammatory cytokines and chemokines, including IL-6, IFN-γ, TNF-α, and CCL-2, were examined. The expression of these pro-inflammatory cytokines and chemokines was significantly decreased in TLR3/dsRNA complex inhibitor treatment group 24 and 36 h after infection (**Figure 6**). In addition, treatment of IAV-infected P815 cells with TLR3/dsRNA complex inhibitor dramatically decreased viral titers 24 and 36 h after infection (**Figure 7**). Taken together, these data suggested that following IAV infection P815 cells actively participated in promoting inflammation by releasing a range of pro-inflammatory cytokines and chemokines possibly through TLR3 signaling pathways.

## Discussion

Elucidating the mechanisms of immune defense against IAV is critical for developing therapeutic strategies to prevent influenza infection. The role of endothelial cells, macrophages, and dendritic cells in preventing infection in the respiratory tract has been described (Bender et al., 1998; Julkunen et al., 2000). However, mast cells, an important cell type in the first lines of host immunity and defense, have been largely overlooked until recently, data from our lab and others have demonstrated a possible involvement of mast cells in IAV infection (Hu et al., 2012; Graham et al., 2013; Marcet et al., 2013). Our previous study showed that mast cells actively participate in the first-line immunological responses to IAV infection. Mast cells could aggravate pathological injury of the H5N1 virus infected tissues in mice by directly inducing apoptosis or inflammatory cytokines and mediators (Hu et al., 2012). However, the receptor repertoire facilitating IAV infection and the cellular response in mast cells are still largely unknown. The present work demonstrated that the mouse mastocytoma cell line (P815) expressed both α-2,3- and α-2,6- linked SA receptors to initiate IAV entry and could serve as a comfortable environment for virus replication and release progeny viruses.

In order to infect cells, IAV must first attach to SA receptors on the plasma membrane. We demonstrated that both α-2,3- and α-2,6- linked SA receptors were expressed on the surface of P815 mouse mastocytoma cells. To our knowledge, this is the first time SA receptors have been reported on the surface of cells considered as mast cells. In addition, our data demonstrated that P815 cells supported the productive replication of IAV. All three subtypes of the viruses (H1N1, H5N1, and H7N2) infected and replicated in P815 cells *in vitro*, with images of transmission electron microscope as the most powerful evidences. Though the mouse mastocytoma cell line, P815, as a cell model, has been widely used in mast cell-based research (Ohtsu et al., 1996; Lunderius et al., 2000; Zhang et al., 2009, 2010), the validity of data from this model should be verified by *in vivo* study or with primary cells. In our present study, the infection of mast cells with H5N1 in mice (*in vivo*) provided further evidence. Contradictory with the results in P815 mouse mastocytoma cells, Marcet’s study showed that H1N1 virus were limited replication in human mast cells (Marcet et al., 2013). Similarly with our results in the present study, several groups showed that dengue virus could infect and replicate within mast cells (St John et al., 2011). Whether mast cells can be infected and serviced as a potent reservoir for persistent Human Immunodeficiency Virus was still a debate until now (Sundstrom et al., 2007). Thus, our findings provided new insights into the role of mast cells for the pathogenesis of influenza.

Activated mast cells can selectively release many pro-inflammatory cytokines and chemokines, which can vary greatly depending on the stimulus and experimental conditions (Abraham and St John, 2010). Here, we found that all the three subtypes of IAVs infected P815 mouse mastocytoma cells produced several cytokines, including a significant increase in the expression of IL-6, IFN-γ, and TNF-α, which could serve as objective markers of the host inflammatory responses and lung injury (Hierholzer et al., 1998; Perrone et al., 2010). Additionally, the chemokines CCL-2 and IP-10 were also increased, which was consistent with observations from both clinical settings and animal models (Zhou et al., 2006). Our results were consistent with a recent report that bone marrow-cultured mast cells infected by the influenza strain A/WSN/33 were found to release several mediators (Graham et al., 2013). Our findings showed that the three subtypes of IAVs induced similar cytokine and chemokine kinetics, and the magnitude of responses induced by H7N2 was much lower than H1N1 and H5N1, which exactly related to the viral replication competence but not the origin of the virus. We also found that the kinetic expression patterns of gene transcription were different for various cytokines and chemokines in P815 cells, some of them were likely directly induced by IAV infection as the time courses of their appearance were early, while others might result from autocrine or paracrine feedback as the late appearance. Interestingly, even though type I interferons are key antiviral cytokines produced by IAV-infected epithelial cells and monocytes/macrophage (Hofmann et al., 1997; Ronni et al., 1997), neither IFN-α nor IFN-β was detected in P815 cells. We speculate that mast cells are involved in the immune and inflammatory responses to AIV infection, but may not directly participate in the antiviral mechanisms.

Mast cells used TLR3, RIG-I and MDA5 to sense viral RNA following infection (Kulka et al., 2004; Oldstone and Rosen, 2014; Becker et al., 2015). In a Newcastle disease virus infection model, mast cells produced cytokines and chemokines in a TLR3-dependent manner (To et al., 2001). In addition, though the degranulation and the generation of eicosanoid in mast cells augmented the vascular leakage and played an important role in dengue virus infection (St John et al., 2013; Syenina et al., 2015), the roles of pattern recognition receptors against the viral infection were also indispensable. Moreover, mast cells infected with dengue virus and vesicular stomatitis virus were shown to activate RIG-I and MDA5, and produce cytokines and chemokines (Jacobs and Langland, 1996; St John et al., 2011; Teijaro et al., 2011; Brown et al., 2012). Importantly, Graham et al. (2013) found the inflammatory response of mast cells during IAV infection occurred in a RIG-I-dependent mechanism. However, the mechanism by which mast cells sense IAV infection is not well understood. Here, for the first time we demonstrated that P815 mouse mastocytoma cells sensed influenza viruses mainly via TLR3. In the present study, we found that blocking TLR3 with TLR3/dsRNA complex inhibitor in the P815 cells resulted in decreased pro-inflammatory factors and viral titers. This data suggested that activated TLR3 pathways were responsible for the production of key pro-inflammatory cytokines and chemokines in IAV infected mast cells. A recent study suggested that TLR3 could act as viral sensor to mediate viral transactivation via upregulation of transcription factors such as c-Jun, which was known to regulate the viral promoter activity (Bhargavan et al., 2016). We recently also showed that IAV infection up-regulated c-jun expression and activation (Xie et al., 2014). This could partially explain that blocking TLR3 resulted in decreased IAV growth.

Collectively, our data suggest that mast cells not only participate in the IAV-induced immune response and inflammation, but also actively serve as reservoirs for IAV replication. Combined with we previously found that mast cells escalated lung injury could be reduced dramatically by treatment with ketotifen, which is a mast cell degranulation inhibitor (Hu et al., 2012; Han et al., 2016). Thus, considering the critical role of mast cells in IAV infection, this study provides insight for the development of novel strategies to combat influenza infection by targeting mast cells.

## Author Contributions

Conceived and designed the experiments: DM, CH, YH, MW, and LS. Performed the experiments: DM, CH, TW, BL, JX, and HD. Analyzed the data: DM, CH, YH, and LS. Contributed reagents/materials/analysis tools: YH, MW, LS, GZ, and HD. Wrote the paper: DM, CH, YH, and LS. All authors reviewed the manuscript.

## Conflict of Interest Statement

The authors declare that the research was conducted in the absence of any commercial or financial relationships that could be construed as a potential conflict of interest.

## References

[B1] Abraham S. N. St John A. L. (2010). Mast cell-orchestrated immunity to pathogens. *Nat. Rev. Immunol.* 10 440–452. 10.1038/Nri2782 20498670 PMC4469150

[B2] Becker M. Lemmermann N. A. W. Ebert S. Baars P. Renzaho A. Podlech J. (2015). Mast cells as rapid innate sensors of cytomegalovirus by TLR3/TRIF signaling-dependent and -independent mechanisms. *Cell. Mol. Immunol.* 12 192–201. 10.1038/Cmi.2014.73 25152077 PMC4654297

[B3] Bender A. Albert M. Reddy A. Feldman M. Sauter B. Kaplan G. (1998). The distinctive features of influenza virus infection of dendritic cells. *Immunobiology* 198 552–567. 10.1016/S0171-2985(98)80078-8 9561373

[B4] Bhargavan B. Woollard S. M. Kanmogne G. D. (2016). Toll-like receptor-3 mediates HIV-1 transactivation via NFkappaB and JNK pathways and histone acetylation, but prolonged activation suppresses Tat and HIV-1 replication. *Cell. Signal.* 28 7–22. 10.1016/j.cellsig.2015.11.005 26569339 PMC4890564

[B5] Brown M. G. McAlpine S. M. Huang Y. Y. Haidl I. D. Al-Afif A. Marshall J. S. (2012). RNA sensors enable human mast cell anti-viral chemokine production and IFN-mediated protection in response to antibody-enhanced dengue virus infection. *PLoS ONE* 7:e34055 10.1371/journal.pone.0034055 PMC3316603 22479521

[B6] Gibbons A. E. Price P. Robertson T. A. Papadimitriou J. M. Shellam G. R. (1990). Replication of murine cytomegalovirus in mast cells. *Arch. Virol.* 115 299–307. 10.1007/BF01310538 2175592

[B7] Graham A. C. Hilmer K. M. Zickovich J. M. Obar J. J. (2013). Inflammatory response of mast cells during influenza A virus infection is mediated by active infection and RIG-I signaling. *J. Immunol.* 190 4676–4684. 10.4049/jimmunol.1202096 23526820 PMC3633673

[B8] Han D. Wei T. Zhang S. Wang M. Tian H. Cheng J. (2016). The therapeutic effects of sodium cromoglycate against influenza A virus H5N1 in mice. *Influenza Other Respir. Viruses* 10 57–66. 10.1111/irv.12334 26176755 PMC4687497

[B9] Hierholzer C. Kalff J. C. Omert L. Tsukada K. Loeffert J. E. Watkins S. C. (1998). Interleukin-6 production in hemorrhagic shock is accompanied by neutrophil recruitment and lung injury. *Am. J. Physiol.* 275 L611–L621. 9728057 10.1152/ajplung.1998.275.3.L611

[B10] Hoffmann E. Stech J. Guan Y. Webster R. G. Perez D. R. (2001). Universal primer set for the full-length amplification of all influenza A viruses. *Arch. Virol.* 146 2275–2289. 10.1007/s007050170002 11811679

[B11] Hofmann P. Sprenger H. Kaufmann A. Bender A. Hasse C. Nain M. (1997). Susceptibility of mononuclear phagocytes to influenza A virus infection and possible role in the antiviral response. *J. Leukoc. Biol.* 61 408–414. 9103226 10.1002/jlb.61.4.408

[B12] Hu Y. Jin Y. Han D. Zhang G. Cao S. Xie J. (2012). Mast cell-induced lung injury in mice infected with H5N1 influenza virus. *J. Virol.* 86 3347–3356. 10.1128/JVI.06053-11 22238293 PMC3302317

[B13] Ibricevic A. Pekosz A. Walter M. J. Newby C. Battaile J. T. Brown E. G. (2006). Influenza virus receptor specificity and cell tropism in mouse and human airway epithelial cells. *J. Virol.* 80 7469–7480. 10.1128/JVI.02677-05 16840327 PMC1563738

[B14] Jacobs B. L. Langland J. O. (1996). When two strands are better than one: the mediators and modulators of the cellular responses to double-stranded RNA. *Virology* 219 339–349. 10.1006/viro.1996.0259 8638399

[B15] Jolly S. Detilleux J. Desmecht D. (2004). Extensive mast cell degranulation in bovine respiratory syncytial virus-associated paroxystic respiratory distress syndrome. *Vet. Immunol. Immunopathol.* 97 125–136. 10.1016/j.vetimm.2003.08.014 14741132

[B16] Julkunen I. Melen K. Nyqvist M. Pirhonen J. Sareneva T. Matikainen S. (2000). Inflammatory responses in influenza A virus infection. *Vaccine* 19 S32–S37. 10.1016/S0264-410X(00)00275-9 11163460

[B17] Kato H. Takeuchi O. Sato S. Yoneyama M. Yamamoto M. Matsui K. (2006). Differential roles of MDA5 and RIG-I helicases in the recognition of RNA viruses. *Nature* 441 101–105. 10.1038/nature04734 16625202

[B18] King C. A. Marshall J. S. Alshurafa H. Anderson R. (2000). Release of vasoactive cytokines by antibody-enhanced dengue virus infection of a human mast cell/basophil line. *J. Virol.* 74 7146–7150. 10.1128/JVI.74.15.7146-7150.2000 10888655 PMC112233

[B19] Knipe D. M. Howley P. M. Griffin D. E. (2001). *Fundamental Virology.* Philadelphia, PA: Lippincott Williams & Wilkins.

[B20] Kulka M. Alexopoulou L. Flavell R. A. Metcalfe D. D. (2004). Activation of mast cells by double-stranded RNA: evidence for activation through toll-like receptor 3. *J. Allergy Clin. Immunol.* 114 174–182. 10.1016/j.jaci.2004.03.049 15241362

[B21] Le Goffic R. Pothlichet J. Vitour D. Fujita T. Meurs E. Chignard M. (2007). Cutting Edge: influenza A virus activates TLR3-dependent inflammatory and RIG-I-dependent antiviral responses in human lung epithelial cells. *J. Immunol.* 178 3368–3372. 10.4049/jimmunol.178.6.3368 17339430

[B22] Lunderius C. Xiang Z. Nilsson G. Hellman L. (2000). Murine mast cell lines as indicators of early events in mast cell and basophil development. *Eur. J. Immunol.* 30 3396–3402. 10.1002/1521-4141(2000012)30:12<3396::AID-IMMU3396>3.0.CO;2-O 11093157

[B23] Marcet C. W. St Laurent C. D. Moon T. C. Singh N. Befus A. D. (2013). Limited replication of influenza A virus in human mast cells. *Immunol. Res.* 56 32–43. 10.1007/s12026-012-8377-4 23055084

[B24] Marshall J. S. (2004). Mast-cell responses to pathogens. *Nat. Rev. Immunol.* 4 787–799. 10.1038/nri1460 15459670

[B25] Matrosovich M. N. Matrosovich T. Y. Gray T. Roberts N. A. Klenk H. D. (2004). Human and avian influenza viruses target different cell types in cultures of human airway epithelium. *Proc. Natl. Acad. Sci. U.S.A.* 101 4620–4624. 10.1073/pnas.0308001101 15070767 PMC384796

[B26] Ohtsu H. Kuramasu A. Suzuki S. Igarashi K. Ohuchi Y. Sato M. (1996). Histidine decarboxylase expression in mouse mast cell line P815 is induced by mouse peritoneal cavity incubation. *J. Biol. Chem.* 271 28439–28444. 10.1074/jbc.271.45.28439 8910469

[B27] Oldstone M. B. A. Rosen H. (2014). Cytokine storm plays a direct role in the morbidity and mortality from influenza virus infection and is chemically treatable with a single sphingosine-1-phosphate agonist molecule. *Curr. Top. Microbiol. Immunol.* 378 129–147. 10.1007/978-3-319-05879-5_6 24728596 PMC7121493

[B28] Perrone L. A. Szretter K. J. Katz J. M. Mizgerd J. P. Tumpey T. M. (2010). Mice lacking both TNF and IL-1 receptors exhibit reduced lung inflammation and delay in onset of death following infection with a highly virulent H5N1 virus. *J. Infect. Dis.* 202 1161–1170. 10.1086/656365 20815704 PMC2941567

[B29] Pichlmair A. Schulz O. Tan C. P. Naslund T. I. Liljestrom P. Weber F. (2006). RIG-I-mediated antiviral responses to single-stranded RNA bearing 5’-phosphates. *Science* 314 997–1001. 10.1126/science.1132998 17038589

[B30] Raman R. Tharakaraman K. Shriver Z. Jayaraman A. Sasisekharan V. Sasisekharan R. (2014). Glycan receptor specificity as a useful tool for characterization and surveillance of influenza A virus. *Trends Microbiol.* 22 632–641. 10.1016/j.tim.2014.07.002 25108746 PMC4252848

[B31] Ronni T. Matikainen S. Sareneva T. Melen K. Pirhonen J. Keskinen P. (1997). Regulation of IFN-alpha/beta, MxA, 2’,5’-oligoadenylate synthetase, and HLA gene expression in influenza A-infected human lung epithelial cells. *J. Immunol.* 158 2363–2374. 9036986

[B32] Shelburne C. P. Abraham S. N. (2011). The mast cell in innate and adaptive immunity. *Adv. Exp. Med. Biol.* 716 162–185. 10.1007/978-1-4419-9533-9_10 21713657

[B33] Shinya K. Ebina M. Yamada S. Ono M. Kasai N. Kawaoka Y. (2006). Influenza virus receptors in the human airway. *Nature* 440 435–436. 10.1038/440435a 16554799

[B34] Shirato K. Taguchi F. (2009). Mast cell degranulation is induced by A549 airway epithelial cell infected with respiratory syncytial virus. *Virology* 386 88–93. 10.1016/j.virol.2009.01.011 19195674

[B35] Springer G. F. Schwick H. G. Fletcher M. A. (1969). Relationship of influenza virus inhibitory activity of glycoproteins to their molecular size and sialic acid content. *Proc. Natl. Acad. Sci. U.S.A.* 64 634–641. 10.1073/pnas.64.2.634 5261039 PMC223391

[B36] St John A. L. Rathore A. P. Raghavan B. Ng M. L. Abraham S. N. (2013). Contributions of mast cells and vasoactive products, leukotrienes and chymase, to dengue virus-induced vascular leakage. *Elife* 2:e00481 10.7554/eLife.00481 PMC3639510 23638300

[B37] St John A. L. Rathore A. P. Yap H. Ng M. L. Metcalfe D. D. Vasudevan S. G. (2011). Immune surveillance by mast cells during dengue infection promotes natural killer (NK) and NKT-cell recruitment and viral clearance. *Proc. Natl. Acad. Sci. U.S.A.* 108 9190–9195. 10.1073/pnas.1105079108 21576486 PMC3107258

[B38] Sundstrom J. B. Ellis J. E. Hair G. A. Kirshenbaum A. S. Metcalfe D. D. Yi H. (2007). Human tissue mast cells are an inducible reservoir of persistent HIV infection. *Blood* 109 5293–5300. 10.1182/blood-2006-11-058438 17351109 PMC1890823

[B39] Sundstrom J. B. Little D. M. Villinger F. Ellis J. E. Ansari A. A. (2004). Signaling through Toll-like receptors triggers HIV-1 replication in latently infected mast cells. *J. Immunol.* 172 4391–4401. 10.4049/jimmunol.172.7.4391 15034054

[B40] Suzuki Y. Ito T. Suzuki T. Holland R. E. Chambers T. M. Kiso M. (2000). Sialic acid species as a determinant of the host range of influenza A viruses. *J. Virol.* 74 11825–11831. 10.1128/Jvi.74.24.11825-11831.2000 11090182 PMC112465

[B41] Syenina A. Jagaraj C. J. Aman S. A. Sridharan A. St John A. L. (2015). Dengue vascular leakage is augmented by mast cell degranulation mediated by immunoglobulin Fcgamma receptors. *Elife* 4:e05291 10.7554/eLife.05291 PMC4362203 25783751

[B42] Takeuchi O. Akira S. (2009). Innate immunity to virus infection. *Immunol. Rev.* 227 75–86. 10.1111/j.1600-065X.2008.00737.x 19120477 PMC5489343

[B43] Teijaro J. R. Walsh K. B. Cahalan S. Fremgen D. M. Roberts E. Scott F. (2011). Endothelial cells are central orchestrators of cytokine amplification during influenza virus infection. *Cell* 146 980–991. 10.1016/j.cell.2011.08.015 21925319 PMC3176439

[B44] Thuy T. B. P. Sugamata R. Uno K. Aratani Y. Ozato K. Kawachi S. (2011). Key role of regulated upon activation normal T-cell expressed and secreted, nonstructural protein1 and myeloperoxidase in cytokine storm induced by influenza virus PR-8 (A/H1N1) infection in A549 bronchial epithelial cells. *Microbiol. Immunol.* 55 874–884. 10.1111/j.1348-0421.2011.00396.x 22039999 PMC4158925

[B45] To K. F. Chan P. K. S. Chan K. F. Lee W. K. Lam W. Y. Wong K. F. (2001). Pathology of fatal human infection associated with avian influenza A H5N1 virus. *J. Med. Virol.* 63 242–246. 10.1002/1096-9071(200103)63:3<242::AID-JMV1007>3.0.CO;2-N 11170064

[B46] Uyeki T. M. (2009). Human infection with highly pathogenic avian influenza A (H5N1) virus: review of clinical issues. *Clin. Infect. Dis.* 49 279–290. 10.1086/600035 19522652

[B47] Wong J. P. Yang H. M. Nagata L. Kende M. Levy H. Schnell G. (1999). Liposome-mediated immunotherapy against respiratory influenza virus infection using double-stranded RNA poly ICLC. *Vaccine* 17 1788–1795. 10.1016/S0264-410X(98)00439-3 10194841

[B48] Xie J. Zhang S. Hu Y. Li D. Cui J. Xue J. (2014). Regulatory roles of c-jun in H5N1 influenza virus replication and host inflammation. *Biochim. Biophys. Acta* 1842(12 Pt. A), 2479–2488. 10.1016/j.bbadis.2014.04.017 24780373

[B49] Yu M. Levine S. J. (2011). Toll-like receptor 3, RIG-I-like receptors and the NLRP3 inflammasome: key modulators of innate immune responses to double-stranded RNA viruses. *Cytokine Growth Factor Rev.* 22 63–72. 10.1016/j.cytogfr.2011.02.001 21466970 PMC3109132

[B50] Zhang H. Lin L. Yang H. Zhang Z. Yang X. Zhang L. (2010). Induction of IL-13 production and upregulation of gene expression of protease activated receptors in P815 cells by IL-6. *Cytokine* 50 138–145. 10.1016/j.cyto.2010.02.006 20189822

[B51] Zhang H. Yang H. Zhang L. Yang X. Zhang Z. Lin Q. (2009). Induction of IL-4 release and upregulated expression of protease activated receptors by GM-CSF in P815 cells. *Cytokine* 48 196–202. 10.1016/j.cyto.2009.07.001 19651524

[B52] Zhou J. F. Law H. K. W. Cheung C. Y. Ng I. H. Y. Peiris J. S. M. Lau Y. L. (2006). Differential expression of chemokines and their receptors in adult and neonatal macrophages infected with human or avian influenza viruses. *J. Infect. Dis.* 194 61–70. 10.1086/504690 16741883 PMC7110244

