# Supplementary material for: Influenza A Viruses Replicate Productively in Mouse Mastocytoma Cells (P815) and Trigger Pro-inflammatory Cytokine and Chemokine Production through TLR3 Signaling Pathway
Source: Front Microbiol. 2017 Jan 12;7:2130. doi: 10.3389/fmicb.2016.02130 (PMC5226950; doi:10.3389/fmicb.2016.02130)
Supplement: Supplementary file 1 [file Data_Sheet_1.pdf]

Influenza A viruses replicate productively in mouse mastocytoma cells (P815) and trigger pro-inflammatory cytokine and chemokine production through TLR3 signaling pathway

Di Meng†, Caiyun Huo†, Ming Wang, Jin Xiao, Bo Liu,Tangting Wei, Guozhong Zhang , Hong Dong ,Yanxin Hu*, Lunquan Sun*,

***Correspondence:** Yanxin Hu：huyx@cau.edu.cn
Lunquan Sun：lunquansun @csu.edu.cn

† **Contributed to the work equally.**

Supplementary Materials and Methods

Hemagglutination (HA) assay

The supernatants of mock or virus-infected cell cultures were harvested at the indicated time points, and HA assays were performed to quantify influenza A virus protein. Virus titers were assessed by the ability to agglutinate red blood cells as previously described(Kawaoka and Neumann, 2012). The assay was carried out in V-bottom microtitre plates. Fifty microliters supernatant samples were serially diluted two-fold using PBS and mixed with an equal volume of 0.5% (v/v) chicken erythrocytes, then incubated for 20-60 min at room temperature. Positive wells formed an adherent, homogeneous layer of erythrocytes, while negative reactions appeared as dots at the bottom of the plates. Viral titers were determined by the highest supernatant dilution giving complete erythrocytes agglutination, and were expressed in hemagglutinating units (HAU).

Plaque assay

Plaque assays were performed to determine virus concentration as previously described(Hu et al., 2012). Briefly, MDCK cell monolayers at >90% confluence in 6-well plates were washed with DMEM and infected with 10-fold serially diluted virus inoculum. After an one hour incubation at 37°C, the inoculum was removed by washing the wells. The cell monolayers were then overlaid with a semisolid agar containing 0.5 µg/ml trypsin tolylsulfonyl phenylalanyl chloromethyl ketone (TPCK) (Sigma, Beijing, China). After plaques developed (approximately 60-72 hours at 37°C and 5% CO2), the cells were fixed and stained with 1% crystal violet. Plaques were counted and the concentration of the initial viral suspension was calculated and expressed in PFU (plaque-forming unit)/ml.Table S1. Target gene primers.

References

Kawaoka, Y., and Neumann, G. (2012). Influenza virus : methods and protocols. New York: Humana.

Hu, Y., Jin, Y., Han, D., Zhang, G., Cao, S., Xie, J., et al. (2012). Mast cell-induced lung injury in mice infected with H5N1 influenza virus. J Virol 86(6), 3347-3356. doi: 10.1128/JVI.06053-11.

Supplemental Data

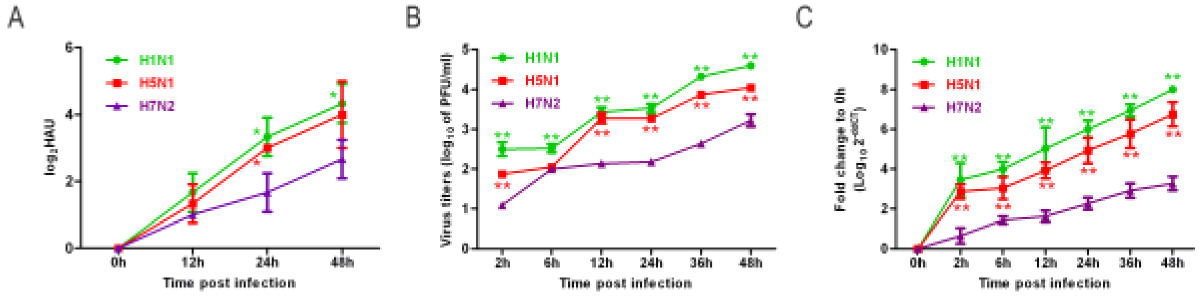

Figure 1S: Influenza viruses can infect P815 cells in vitro. P815 cells were mock-treated or infected with three subtypes of influenza viruses at a MOI of 0.1. Culture supernatants were collected at the indicated times post-infection, and viral protein quantification and virus titers in the supernatants were determined by Hemagglutination (HA) assay (left) and standard plaque assay (middle). Cells were homogenized in Trizol and relative viral NS gene quantification was determined by real time PCR (right). Results shown are pooled from three independent repeats. The viral loads of H1N1 (green) or H5N1 (red) were significantly higher than H7N2 (*P＜0.05 and **P＜0.01) respectively.

Table S1. Target gene primers.

Target gene	Primers
β-actin	5' - GAG ACC TTC AAC ACC CCA GC - 3'
	5' - ATG TCA CGC ACG ATT TCC C - 3'
NS	5' - GCA ATT GGA ATC CTC ATC GG - 3'
	5' - CAA CTC GTT TCG CCA TGT AGC - 3'
TLR-3	5' - CCC TTC ACC TTT CCG - 3'
	5' - TCA TCT AAG CCG TTG G - 3'
RIG-I	5' - TTC TAA AGC CTG GCA TAC TGA - 3'
	5' - GCT ATC TCG TGC TCT TCC TC - 3'
MDA-5	5' - CTT CCT GGA TGT TCT GCG CCA A - 3'
	5' - CCG TGG GGA GGC AGA TAA TAA T - 3'
TRIF	5' - AAC CTC CAC ATC CCC TGT TTT - 3'
	5' - CGG GCA CCT GAA ATT CCT CA - 3'
MAVS	5' - GCT GTA CCG TAG TAG GCG - 3'
	5' - GTC AGG AGC AAT GGA GG - 3'
IL-1α	5' - CAC CTT ACA CCT ACC AGA GTG ATT TG - 3'
	5' - TGT TGC AGG TCA TTT AAC CAA GTG - 3'
IL-1β	5' - TCC AGG ATG AGG ACA TGA GCA C - 3'
	5' - GAA CGT CAC ACA CCA GCA GGT TA - 3'
IL-6	5' - AGC CAG AGT CCT TCA - 3'
	5' - TCT TGG TCC TTA GCC - 3'
IL-10	5' - GGT TGC CAA GCC TTA TCG GA - 3'
	5' - ACC TGC TCC ACT GCC TTG CT - 3'
IL-12	5' - CAA CAT CAA GAG CAG TAG CAG - 3'
	5' - TAC TCC CAG CTG ACC TCC AC - 3'
IL-13	5' - GCT TAT TGA GGA GCT GAG CAA CA - 3'
	5' - GCC AGG TCC ACA CTC CAT A - 3'
IFN-α	5' - GGA CTT TGG ATT CCC GCA GGA GAA G - 3'
	5' - GCT GCA TCA GAC AGC CTT GCA GGT C - 3'
IFN-β	5' - AAC CTC ACC TAC AGG GCG GAC TTC A - 3'
	5' - TCC CAC GTC AAT CTT TCC TCT TGC TTT - 3'
IFN-γ	5' - ACA CTG CAT CTT GGC TTT GCA GCT - 3'
	5' - TGA GCT CAT TGA ATG CTT GGC GCT - 3'
TNF-α	5' - CTG TAG CCC ACG TCG TAG C - 3'
	5' - TTG AGA TCC ATG CCG TTG - 3'
TGF-β	5' - AAG GGA AAG CAT GAA TGG AGC GCT - 3'
	5' - TCA AGC TCT TTG CCT TGC CCT GAA - 3'
IP-10	5' - GTC CGC TGC AAC TGC ATC CAT A - 3'
	5' - CTG CTC ATC ATT CTT TTT CAT CGT G - 3'
CCL2/MCP-1	5' - CAC CAT GCA GGT CCC TGT CAT G - 3'
	5' - GAT CTC ATT TGG TTC CGA TCC A - 3'

CCL3/MIP-1α	5' - ACT GCC TGC TGC TTC TCC TAC A - 3'
	5' - AGG AAA ATG ACA CCT GGC TGG - 3'
CCL4/MIP-1β	5' - AAA CCT AAC CCC GAG CAA CA - 3'
	5' - CCA TTG GTG CTG AGA ACC CT - 3'
CCL5/RANTES	5' - GCC CAC GTC AAG GAG TAT TTC TAC - 3'
	5' - AGG ACT AGA GCA AGC GAT GAC AG - 3'
CCL12/MCP-5	5' - TGG CTG GAC CAG ATG CG - 3'
	5' - GAC GTG AAT CTT CTG CTT AAC AAC A - 3'
CXCL1/KC	5' - CAC AGG GGC GCC TAT CGC CAA - 3'
	5' - CAA GGC AAG CCT CGC GAC CAT - 3'
CXCL2/MIP-2	5' - TGC CGG CTC CTC AGT GCT G - 3'
	5' - AAA CTT TTT GAC CGC CCT TGA - 3'
CXCL10/IP-10	5' - GTC CGC TGC AAC TGC ATC CAT A - 3'
	5' - CTG CTC ATC ATT CTT TTT CAT CGT G - 3'

Table S2. mRNA profile of cytokine and chemokine gene expressions of LE-PolyI:C- treated or viruses-infected P815 cells at indicated time points.

	Fold change to mock																							
	polyI:C						H1N1						H5N1						H7N2					
**Genes**	**2h**	**6h**	**12h**	**24h**	**36h**	**48h**	**2h**	**6h**	**12h**	**24h**	**36h**	**48h**	**2h**	**6h**	**12h**	**24h**	**36h**	**48h**	**2h**	**6h**	**12h**	**24h**	**36h**	**48h**
**IL-1α**	1.7±0.23	2.6±0.38	**4.6±0.15[b]**	**15±0.48[b]**	**3.7±0.21[b]**	3.0±0.12[b]	2.1±0.082[b]	**3.1±0.09[b]**	**19±1.8[b]**	**21±1.3[b]**	2±0.46	**6.6±0.74[a]**	2.4±0.23[b]	**3.5±0.24[b]**	**21±0.43[b]**	**4.4±0.56[a]**	**3.8±0.25[b]**	**19±1.7[b]**	1.6±0.13	2.6±0.21[a]	**4.6±0.42[b]**	**11±1.3[a]**	**4.9±0.057[b]**	**12±0.47[b]**
**IL-1β**	0.68±0.15	1.7±0.32	2.6±0.29[a]	2.5±0.12[b]	2.5±0.045[b]	1.3±0.14	2.5±0.42	**4.0±0.62[a]**	**4.8±0.42[b]**	**3.4±0.2[b]**	**13±0.81[b]**	**15±1.1[b]**	**3.5±0.52[a]**	**5.1±0.22[b]**	**6.9±0.11[b]**	1.4±0.028[b]	**6.0±0.36[b]**	**14±0.49[b]**	1.2±0.3	1.8±0.25	2.3±0.16[a]	2.1±0.084[b]	**4.3±0.036[b]**	**3.3±0.15[b]**
**IL-6**	**17±4.2**	**22±3.9[a]**	**33±4.8[a]**	**80±3.4[b]**	**35±7.7**	**20±3.0[a]**	**13±2.6[a]**	**12±1.3[b]**	**16±2.1[a]**	**37±0.90[b]**	**58±6.4[b]**	**70±5.3[b]**	**9.2±0.91[b]**	**10±1.5[a]**	**10±1.3[a]**	**28±3.8[a]**	**21±3.6[a]**	**15±1.9[a]**	**9.5±1.6[a]**	**6.9±1.2[a]**	**6.8±1.1[a]**	**13±2.3[a]**	**21±3.8[a]**	**33±6.1[a]**
**IL-10**	1.23±0.33	2.78±0.04[a]	**4.03±0.45[b]**	**10.1±0.67[b]**	1.49±0.07[a]	0.96±0.06	1.00±0.25	1.02±0.15	1.10±0.27	1.05±0.34	1.09±0.07	0.92±0.10	1.02±0.22	1.03±0.17	1.13±0.09	0.95±0.18	1.15±0.10	0.98±0.13	1.02±0.19	1.11±0.22	0.94±0.07	0.99±0.13	1.00±0.14	1.02±0.13
**IL-12**	1.8±0.16[a]	1.8±0.18	**3.7±0.77**	**9.5±0.82[b]**	1.4±0.059[a]	1.6±0.062[b]	1.6±0.12[a]	**3.6±0.56[a]**	**14±0.82[b]**	**12±1.1[b]**	**5.3±0.76[a]**	**4.1±0.27[b]**	1.4±0.14	2.2±0.37	**6.9±0.17[b]**	1.9±0.34	0.56±0.06[a]	0.47±0.02[b]	1.7±0.096[a]	1.8±0.11[a]	**4.6±0.13[b]**	**5.5±0.25[b]**	1.8±0.018[b]	1.6±0.26
**IL-13**	**3.2±0.17[b]**	**11±1.6[a]**	**48±0.44[b]**	**15±0.54[b]**	0.46±0.03[b]	0.08±0.002[b]	2.9±0.12[b]	**9.6±0.96[b]**	**38±2.7[b]**	**29±0.47[b]**	1.3±0.025[b]	1.0±0.04	2.1±0.13[a]	**4.5±0.33[b]**	**14±0.92[b]**	2.8±0.076[b]	0.99±0.04	0.70±0.04[a]	1.6±0.056[b]	2.0±0.11[b]	2.5±0.12[b]	2.5±0.073[b]	0.58±0.02[b]	0.54±0.03[b]
**IFN-α**	2.6±0.048[a]	**3.8±0.045[a]**	**5.03±0.19[b]**	**10.4±0.71[b]**	2.29±0.06[a]	1.11±0.01	0.94±0.11	0.95±0.20	1.03±0.12	1.00±0.17	1.07±0.19	1.04±0.16	1.04±0.15	1.05±0.12	0.99±0.16	1.06±0.09	0.98±0.15	1.01±0.12	1.00±0.12	1.06±0.19	0.99±0.08	1.05±0.13	1.04±0.16	0.98±0.16
**IFN-β**	1.11±0.064	1.89±0.13	2.5±0.25[a]	**4.2±0.05[b]**	1.4±0.03	0.96±0.02	0.95±0.11	0.97±0.20	1.05±0.21	0.96±0.16	1.04±0.10	1.01±0.14	1.02±0.13	1.03±0.19	0.98±0.11	1.02±0.06	0.96±0.16	1.03±0.20	0.95±0.14	1.01±0.05	0.97±0.09	0.98±0.19	1.02±0.09	1.03±0.15
**IFN-γ**	2.3±0.48	**3.3±0.69**	**14±1.7[a]**	**22±1.1[b]**	**43±5.1[a]**	**110±2.5[b]**	2.5±0.63	**4.0±0.71**	**9.8±0.65[b]**	**16±1.6[b]**	**25±2.1[b]**	**30±4.4[a]**	2.5±0.51	**8.0±1.3[a]**	**15±2.6[a]**	**27±1.8[b]**	**89±12[a]**	**93±7.7[b]**	1.5±0.33	**3.6±0.30[a]**	**5.6±1.9**	**12±4.8**	**10±2.4**	**18±4.5**
**TNF-α**	0.52±0.084[b]	1.3±0.14	**6.1±1.3**	**20±1.2[b]**	0.67±0.12	1.1±0.37	2.1±0.29	2.6±0.21[a]	**4.7±1.2**	**11±1.3[a]**	**8.0±0.65[b]**	**3.9±1.3**	2.2±0.20[a]	2.8±0.20[b]	**6.4±0.67[a]**	**14±1.8[a]**	**5.7±0.94[a]**	**4.0±0.80**	1.1±0.093	1.7±0.10[a]	2.4±0.073[b]	**4.7±0.31[b]**	2.3±0.21	1.3±0.27
**TGF-β**	0.94±0.16	0.75±0.078	0.81±0.06	1.2±0.067	0.75±0.08	**4.7±0.56[a]**	**6.1±0.49[b]**	**4.8±0.38[b]**	**4.8±0.18[b]**	2.9±0.19[b]	**11±1.5[a]**	**112±5.6[b]**	0.96±0.054	0.90±0.10	1.3±0.076	0.97±0.09	**14±3.8**	**91±5.5[b]**	0.48±0.019[b]	0.35±0.028[b]	0.91±0.05	1.1±0.24	**3.5±0.63**	**13±1.9[a]**
**CCL2**	1.7±0.23	2.8±0.14[b]	**4.2±0.12[b]**	**4.7±0.36[b]**	**6.2±0.35[b]**	**8.1±0.44[b]**	1.1±0.066	1.6±0.11[a]	**3.3±0.20[b]**	**4.4±0.075[b]**	**5.5±0.086[b]**	**5.9±0.11[b]**	1.1±0.066	1.5±0.039[a]	2.9±0.16[b]	**3.6±0.37[a]**	**4.0±0.43[a]**	**5.2±0.51[a]**	1.2±0.025	1.6±0.15	2.1±0.098[b]	2.7±0.11[b]	**3.1±0.15[b]**	**4.0±0.20[a]**
**CCL3**	**4.9±0.65[a]**	**9.8±1.4[a]**	**7.7±1.4[a]**	**7.1±0.43[b]**	**8.9±1.7[a]**	**9.9±3.3**	1.8±0.20	**4.8±0.49[a]**	**6.8±1.3**	**4.9±0.66[a]**	**4.1±1.1**	**239±19[b]**	**7.0±1.9**	**30±1.1[b]**	**28±6.7**	**4.2±1.0**	**21±8.8**	**142±9.4[b]**	1.8±0.28	**4.7±1.9**	**3.7±1.2**	**4.6±1.7**	**9.2±4.4**	**30±8.2**
**CCL4**	**4.1±0.052[b]**	**10±0.92[b]**	**31±2.1[b]**	**13±0.63[b]**	0.34±0.04[b]	0.67±0.44	1.3±0.095	**3.7±0.55[a]**	**21±2.3[b]**	**14±0.36[b]**	2.1±0.12[b]	0.96±0.15	**12±2.1[a]**	**15±2.3[a]**	**49±4.7[b]**	**7.3±0.85[a]**	**7.0±2.4**	**8.2±1.9**	1.3±0.22	1.6±0.19	**5.1±0.42[b]**	**3.1±0.45[a]**	1.2±0.18	0.48±0.25
**CCL5**	1.1±0.049	1.1±0.12	1.2±0.071	1.1±0.075	0.95±0.07	0.89±0.01[a]	0.97±0.029	1.0±0.024	1.0±0.048	1.6±0.1[a]	1.8±0.14[a]	1.5±0.16[a]	0.95±0.047	0.98±0.073	0.78±0.04	0.9±0.079	1.2±0.045	1.1±0.12	0.81±0.040	0.90±0.12	0.57±0.05[a]	0.79±0.03	1.0±0.12	0.92±0.14
**CCL12**	0.61±0.068[a]	**3.2±0.51**	**12±0.74[b]**	1.5±0.081[a]	0.09±0.01[b]	0.2±0.009[b]	0.86±0.021	2.1±0.28	**8.1±0.082[b]**	1.2±0.071	0.23±0.04[b]	0.63±0.03[a]	1.1±0.063	1.6±0.061[b]	**6.5±0.35[b]**	0.82±0.05	0.42±0.07[a]	1.5±0.09[a]	0.14±0.046[b]	1.0±0.081	1.4±0.14	0.33±0.01[b]	0.35±0.03[b]	0.28±0.06[b]
**CXCL1**	1.2±0.049	3.0±0.084[b]	**6.6±0.57[b]**	**18±0.43[b]**	1.4±0.11	0.53±0.12	1.0±0.086	1.6±0.13	**3.8±0.49[a]**	1.7±0.045[a]	0.88±0.05	**15±1.6[b]**	2.2±0.28	2.2±0.13[b]	**9.2±1.2[a]**	0.32±0.09[a]	0.49±0.03[b]	2.5±0.37	1.1±0.11	2.1±0.31	**4.0±0.079[b]**	**9.9±0.41[b]**	**6.9±0.37[b]**	**14±1.4[b]**
**CXCL2**	0.63±0.094	1.4±0.19	2.7±0.29[a]	2.9±0.51	0.5±0.053[a]	0.25±0.04[b]	0.94±0.066	0.99±0.049	1.0±0.040	0.69±0.01[b]	1.6±0.21	**7.4±0.44[b]**	0.58±0.080	1.0±0.054	0.48±0.10[a]	0.57±0.03[b]	2.1±0.19[a]	**7.5±0.70[b]**	0.4±0.059[a]	0.53±0.060[a]	0.54±0.06[a]	0.75±0.14	0.28±0.04[b]	0.34±0.08[a]
**CXCL10**	0.75±0.13	1.5±0.38	**4.3±0.99**	1.7±0.29	0.007±0.001[b]	0.006±0.002[b]	0.36±0.048[b]	0.53±0.27	**6.5±0.94[a]**	0.92±0.08	0.86±0.05	0.33±0.08[a]	0.55±0.042[a]	1.4±0.39	**6.2±1.2**	1.8±0.35	1.5±0.40	0.47±0.14	0.86±0.083	1.5±0.27	**3.5±0.64**	0.77±0.30	0.18±0.07[a]	0.02±0.02[b]

Values were normalized to housekeeping gene β-actin and expressed as relative fold change (Mean ± SEM (standard error of mean)) to mock-treated cells.
Bold and red numbers indicated ≥ 3-fold higher levels of mRNA for the respective gene over that of mock infection.
[a] $P<0.01$ and [b] $P<0.01$
